# Supplementary material for: One-Pot Method of Synthesizing TEMPO-Oxidized Bacterial Cellulose Nanofibers Using Immobilized TEMPO for Skincare Applications
Source: Polymers (Basel). 2019 Jun 14;11(6):1044. doi: 10.3390/polym11061044 (PMC6631351; doi:10.3390/polym11061044)
Supplement: Supplementary file 1 [file polymers-11-01044-s001.pdf]

Supporting information

One-Pot Method of Synthesizing TEMPO-Oxidized Bacterial Cellulose Nanofibers Using Immobilized TEMPO for Skincare Applications

Seung-Hyun Jun [1], Sun-Gyoo Park [1] and Nae-Gyu Kang [1,*]

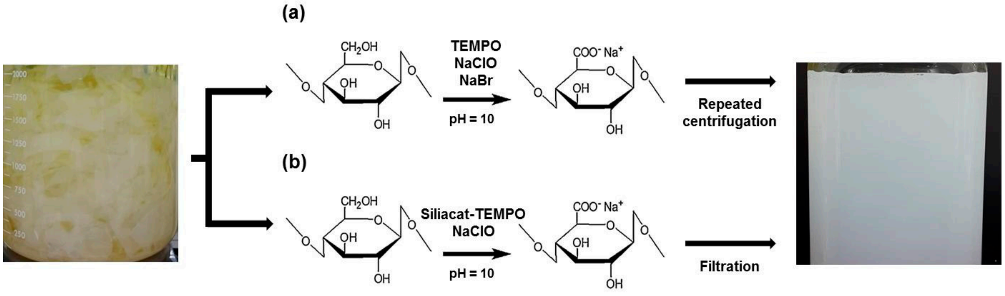

Figure S1. Schematic showing the synthesis of TOCNs via the (a) conventional process and (b) the one-pot process.

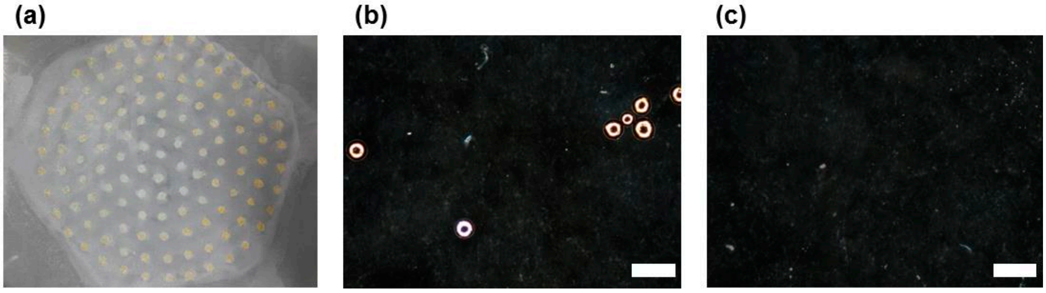

Figure S2. (a) Filtrated TEMPO-immobilized silica beads on the nylon mesh after the oxidation reaction, and the immobilized TEMPO, sodium hypochlorite, and BC solution (b) before and (c) after filtration. Scale bar = 300 μm.

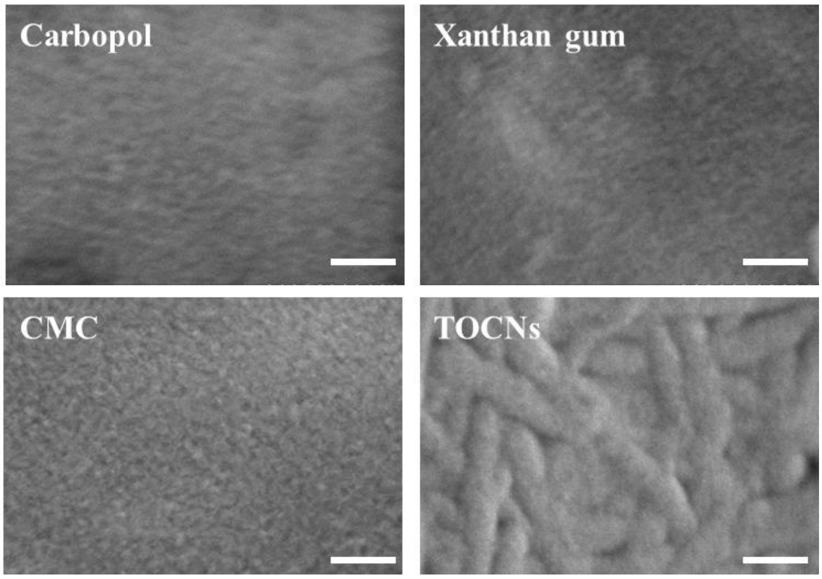

Figure S3. Nanometer-resolution structures of the TOCNs and other bio-polymers obtained via high-resolution imaging using a FE-SEM. Scale bar = 100 nm.
